# Prevalence of post-traumatic stress and tests of metacognition as a PTSD risk marker in patients with coronary heart disease and elevated HADS scores: analysis of data from the PATHWAY RCT's in UK cardiac rehabilitation

**DOI:** 10.3389/fpsyt.2023.1198202

**Published:** 2023-07-06

**Authors:** Adrian Wells, Calvin Heal, David Reeves, Lora Capobianco

**Affiliations:** ^1^Division of Psychology and Mental Health, School of Psychological Sciences, Faculty of Biology, Medicine and Health, The University of Manchester, Manchester, United Kingdom; ^2^Research and Innovation, Greater Manchester Mental Health NHS Trust, Manchester, United Kingdom; ^3^Centre for Biostatistics, Faculty of Biology, Medicine and Health, Manchester Academic Health Sciences Centre, University of Manchester, Manchester, United Kingdom; ^4^NIHR School for Primary Care Research, Manchester Academic Health Sciences Centre, University of Manchester, Manchester, United Kingdom

**Keywords:** coronary heart disease, post-traumatic stress disorder (PTSD), metacognition, cardiac rehabilitation, metacognitive model, IES-R, metacognitions questionnaire, MCQ-30

## Abstract

**Introduction:**

Anxiety and depression in coronary heart disease (CHD) are associated with poorer health outcomes, greater healthcare use and reduced quality of life. Post-traumatic stress symptoms may be a particular concern as they are associated with increased mortality at follow-up. We examined prevalence of PTSD in patients with elevated anxiety/depression scores referred for cardiac rehabilitation (CR) across seven NHS sites in North-West England. We tested a possible mechanism (metacognition) linking CHD to PTSD symptom severity as implicated in the metacognitive model.

**Methods:**

Data was collected at baseline as part of the NIHR funded PATHWAY trial of metacognitive therapy for anxiety and depression in CHD. Patients (*n* = 572) with at least mild symptoms of anxiety and depression under routine screening (assessed with the Hospital Anxiety and Depression Scale) and attending CR were eligible for the study. A battery of questionnaires, including assessment of demographic variables, PTSD symptoms (using the IES-R) and metacognitive beliefs was administered prior to random allocation and intervention delivery.

**Results:**

Rates of PTSD were high, with 48% of patients meeting threshold for PTSD and a further 15% partial PTSD. All five metacognition subscales were positively associated with PTSD vs. no PTSD, with beliefs about the uncontrollability and danger of worry and beliefs about need to control thoughts being most strongly related. For every unit increase in uncontrollability and danger metacognitions the odds of being in the PTSD group increased 30%, whilst the odds of partial PTSD increased 16%. Stepwise regression analysis using the metacognitive subscales along with demographic and health-related covariates found that uncontrollability/danger and need for control metacognitions explained unique variation in PTSD symptom severity, with unique contributions also for age, sex, and number of comorbidities.

**Conclusion:**

PTSD symptoms appeared highly prevalent in the current CR sample. Metacognitive beliefs were individually associated with symptom severity with the strongest positive relationship observed for beliefs about uncontrollability and dangerousness of worry, followed by need to control thoughts. The results highlight the importance in assessing PTSD in CR patients and add support to implementing metacognitive therapy in CHD to target particular metacognition risk factors in anxiety, depression and PTSD.

## Introduction

At least 1 in 3 coronary heart disease (CHD) patients experience anxiety and depression symptoms and anxiety and depression appear to be independent risk factors associated with the development and recovery from CHD (1, 2). Detailed assessment of psychological morbidity amongst cardiac rehabilitation patients suggests that as many as 45% could meet diagnostic criteria for a specific anxiety disorder, 20% could meet criteria for depression with 26% having at least two disorders (3). Additionally, in one study, the early age myocardial infarction (MI) group, showed rates of current psychiatric disorders, lifetime psychiatric disorders, and lifetime depressive disorders that were higher than the rates for the late age MI group (4).

The prevalence of PTSD in CHD has received particular attention, with rates estimated at between 0–35% (5). A meta-analysis of 13 studies involving 821 individuals after myocardial infarction (MI) concluded that the weighted prevalence of PTSD is 14% (range 0–25%) of patients (6). Estimates of PTSD in survivors of MI have been reported as 27% at a mean of 45 months after cardiac arrest (7). A substantial proportion of patients with implantable cardioverter defibrillators (ICDs) appear to have PTSD (8, 9) with a prevalence found to be 20% in ICD clinics (10). PTSD symptoms are a concern because they may be associated with increased mortality and morbidity in CHD (11), but they are not usually assessed or treated as part of cardiac rehabilitation (CR).

Following a cardiovascular event such as MI, clinical guidelines in the UK recommend that patients attend cardiac rehabilitation to reduce future cardiac risk and improve outcomes (12). In 2019 there were 89,573 patients taking up CR across 230 programmes, representing 50% of those offered CR (13). Routine assessment of psychological functioning in CR uses the Hospital Anxiety and Depression Scale (HADS) to assess anxiety and depression symptoms, but this is not designed to capture symptoms of PTSD (re-experiencing, hyperarousal, avoidance). As a result, routine psychological assessment is not geared to recognize PTSD symptoms and adjust to the needs of traumatized patients. An important issue therefore, concerns the prevalence and severity of PTSD in those patients who show at least mild general anxiety/depression symptoms under routine screening and whether greater attention to PTSD is indicated.

In the present study we aimed to assess the magnitude and prevalence of PTSD symptoms in patients endorsing at least mild anxiety/depression symptoms on the routinely administered Hospital Anxiety and Depression Scale (HADS). We also aimed to test the statistical predictors of PTSD symptoms based on metacognitive theory whilst controlling for known covariates. In particular, we examined age, sex, number of comorbidities as covariates and tested the contribution of hypothesized psychological mechanisms which are vulnerabilities for PTSD in the metacognitive model (14). The examination of theory-driven psychological mechanisms that could be risk markers for PTSD is valuable as it might contribute to treatment planning.

The metacognitive model of PTSD (14, 15) links traumatic experiences such as cardiac events to PTSD through the mechanism of excessive negative thinking caused by maladaptive metacognition. It proposes that acute stress symptoms are maintained by activation of a persistent thinking style dominated by worry/rumination, threat monitoring and counterproductive coping behaviors, that maintain a sense of current threat. This thinking style is modulated by metacognition; an array of higher-level structures and internal information involved in the regulation of thinking. An important feature of metacognition in this model is information about cognition on which processing relies. In particular, maladaptive knowledge or beliefs about the uncontrollability and dangerousness of thoughts are considered especially important in the unhelpful regulation of thinking and development of psychological disorder, including PTSD symptoms. Consistent with this prediction, maladaptive metacognitions have been found to be elevated across psychological disorders (16) and among individuals with PTSD (17, 18). A small number of studies suggest that such metacognitions are positively associated with greater anxiety and depression (19) and greater negative affectivity (20) in CHD, and poorer mental health in Pulmonary Arterial Hypertension (21). However, there is a gap in the literature concerning the contribution of metacognitive beliefs to PTSD in CHD patients.

Given these limitations in the literature and the potential value in understanding the extent and risk factors of PTSD in CR the present study aimed to assess: (1) the severity of PTSD symptoms and prevalence of PTSD in CR patients reporting elevated anxiety/depression; (2) the relative predictors of PTSD symptoms; (3) the contribution of biased metacognitions to risk and symptom severity as hypothesized by metacognitive theory, when controlling for covariates. Addressing such issues can contribute to understanding the specific psychological needs of CR patients, support the tailoring of psychological provision to meet those needs and provide evidence of the potential contribution metacognition can make in predicting the risk of PTSD in heart disease patients with elevated anxiety/depression scores.

## Methods

### Participants and design

The present study is a secondary analysis of data from 572 participants (363 males) who participated in the NIHR-funded PATHWAY programme, which was conducted across seven CR services in the North-West of England. Participants completed baseline assessments prior to randomization in two separate controlled trials. Characteristics of sample participants are displayed in Table 1. The trials were single-blind multi-center tests of the effects of adding metacognitive therapy group treatment (trial 1) or adding self-help metacognitive therapy (trial 2) to usual CR (22, 23).

**Table 1:** Participant characteristics.

**Characteristic**	**Total**	**No PTSD**	**Partial PTSD**	**PTSD**
Sex, *n* (%)				
Male	363 (63%)	153 (73%)	55 (65%)	155 (56%)
Female	209 (37%)	58 (27%)	30 (35%)	121 (44%)
Age, mean (SD)	60.5 (10.9)	63.1 (11.0)	61.3 (9.9)	58.2 (10.5)
Previous anxiety	190 (33%)	41 (19%)	28 (33%)	121 (44%)
Previous depression	190 (33%)	42 (20%)	30 (35%)	118 (43%)
Number of comorbidities, mean (SD)	4.6 (2.3)	4.1 (2.0)	4.5 (2.4)	5.0 (2.4)
0	15 (3%)	5 (2%)	3 (4%)	7 (3%)
1–3	193 (34%)	83 (39%)	29 (34%)	81 (29%)
4–6	244 (43%)	98 (46%)	33 (39%)	113 (41%)
7 or more	120 (21%)	25 (12%)	20 (24%)	75 (27%)
Number of additional cardiac events, mean (SD)	0.7 (1.1)	0.7 (1.0)	0.7 (1.0)	0.7 (1.1)
0	333 (58%)	119 (25%)	48 (56%)	166 (60%)
1	122 (21%)	52 (25%)	20 (24%)	50 (18%)
2 or more	117 (21%)	40 (19%)	17 (20%)	60 (22%)
Marital status				
In a relationship	343 (60%)	138 (65%)	46 (54%)	159 (58%)
Separated	132 (23%)	46 (22%)	22 (26%)	64 (23%)
Single	96 (17%)	27 (13%)	17 (20%)	52 (19%)
MCQ-30, mean (SD)	61.7 (15.5)	53.4 (13.3)	61.0 (13.0)	68.2 (14.7)
Positive subscale	10.6 (4.4)	9.4 (3.8)	10.7 (4.6)	11.5 (4.5)
Negative (ud) subscale	13.1 (4.6)	10.4 (3.7)	12.9 (3.9)	15.1 (4.3)
cc subscale	11.7 (5.0)	10.3 (4.3)	11.3 (4.7)	12.8 (5.2)
nfc subscale	11.8 (3.9)	10.4 (3.6)	11.2 (3.2)	13.1 (3.9)
csc subscale	14.5 (4.2)	12.9 (4.0)	14.9 (4.0)	15.6 (4.0)
IES-R, mean (SD)	32.1 (18.7)	12.7 (6.6)	27.7 (2.6)	48.3 (11.4)

MCQ, metacognitions questionnaire; ud, uncontrollability and danger; cc, cognitive confidence; nfc, need for control; csc, cognitive self confidence; IES-R, Impact of Events Scale-Revised.

Eligible patients were referred to CR services and met the Department of Health or British Association for Cardiac Prevention and Rehabilitation CR eligibility criteria. Reasons for referral to CR were: acute coronary syndrome, after revascularization, stable heart failure, stable angina, after implantation of defibrillator, heart valve repair/replacement, heart transplant, and ventricular assist devices, adult congenital heart failure. Patients were required to have a minimum score of 8 (mild symptoms) on either the depression or anxiety subscale of the Hospital Anxiety and Depression Scale (HADs) (24), be aged 18 years or older, and have a competent level of English language comprehension (read, understand, and complete questionnaires in English).

### Outcome measures

At baseline assessment a battery of self-report measures was used. Included in the test battery and extracted for the present analyses were the following measures:

The Impact of Event Scale – Revised (IES-R) (25) is a 22-item self-report measure that assesses symptoms of PTSD, consistent with diagnostic criteria of DSM -IV (26), and is an extension of the IES. Items can be scored as a total score (0–88) or utilize the three subscales: avoidance, intrusion and hyperarousal. The IES-R can be used as a continuous measure and can also be considered using cut-offs to suggest clinical presence of PTSD, where scores 33 or above have been found to give the best diagnostic accuracy for probable PTSD, (27). Partial PTSD scores range between 24 and 32 or the absence of PTSD scores below 24 (28, 29). Although the IES was not originally designed as a diagnostic tool it has excellent sensitivity and specificity in identifying PTSD cases against diagnostic DSM-IV and ICD-10 screening (30). Similarly, the IES-R shows moderate agreement with a positive PTSD screen on DSM-IV and DSM-V (31). In the current sample the Cronbach alpha for the IES-R = 0.95.

The Hospital Anxiety and Depression Scale (24) is a 14-item self-report measure that assesses symptoms of anxiety and depression, is widely used in physical health and is a routine measure of anxiety and depression in CR in the UK. In the present study, Cronbach alpha for the anxiety subscale = 0.82 and for depression = 0.78.

The metacognitions Questionnaire 30 (32) is a 30-item measure of metacognitive beliefs implicated in the metacognitive model. It consists of 5 subscales, assessing the following metacognitive domains; positive beliefs about worry (Pos); negative beliefs about uncontrollability and danger of thoughts (Neg ud); need for control of thoughts (nfc); cognitive confidence (cc), and cognitive self-consciousness (csc). Each subscale is comprised of 6-items. The measure has been widely used in research testing the metacognitive model and has a stable factor structure and acceptable reliability. In CR patients the five factor and a bi-factor model (i.e., including the total score) have been tested for goodness of fit. Whilst it appears that the bi-factor solution may carry some small additional information beyond the 5 subscales alone, continued use of the more widely established 5-factors is currently recommended (18). The subscale Cronbach alpha's in the present sample were as follows: positive beliefs (Pos) = 0.87; Neg ud = 0.82; cc = 0.89; nfc = 0.71; csc = 0.78; MCQ total-score alpha = 0.90.

### Data analysis plan

Descriptive statistics (frequencies and percentages) were used to assess the range and distribution of PTSD symptom severity scores. Prevalence of PTSD was assessed using the IES-R cut-off scores as defined above for no PTSD, partial PTSD and PTSD case-ness. Scatter plots and Pearson's correlation coefficients were used to test associations between metacognitive beliefs and PTSD symptoms (continuous outcome).

To assess if metacognitive beliefs predicted PTSD caseness (defined as: no PTSD, partial, yes), we conducted a multinomial regression. Along with the measures of metacognitions this analysis included a pre-specified set of demographic- and health-related covariates known to be associated with PTSD: age, sex, previous cardiovascular events (yes/no), number of comorbidities and previous diagnosis/treatment of anxiety (yes/no), and/or depression (yes/no). For this analysis we report the relative risk ratios (RR) for the raw subscale and total MCQ scores but we also rescaled the MCQ total and subscale scores to all range from 0–100, to provide directly comparable measures of relationships with PTSD.

To investigate relationships between metacognitive beliefs and degree of PTSD symptomology we conducted a series of linear regression models using IES-R in its continuous form as the dependent variable. An initial “reference” model consisted solely of the pre-specified covariates. Subsequent models included the covariate set plus one MCQ-30 subscale of interest, repeating until the total score and each subscale had been tested individually. Likelihood ratio tests were used to determine if the change in the adjusted R-squared when adding each MCQ-30 subscale score into the model was statistically significant.

We also sought to determine the subset of MCQ subscales which, after controlling for the covariate set could account for unique variation in trauma-symptom severity (IES-R) using hierarchical multiple regressions. To do so we determined whether forward-stepwise regression and backward-stepwise regressions gave rise to the same final subset of MCQ predictors and covariates. All analysis was conducted using Stata version 14 and an alpha level of 5% for statistical significance.

## Results

### Sample overview

Participant characteristics are summarized in Table 1 for the entire sample and are further broken down by PTSD classification based on IES-R cut-offs. The table presents descriptives for the covariates and the MCQ30 subscales.

### Prevalence of PTSD in cardiac rehabilitation patients

While 211 patients (36.9%) did not meet the cut-off score for PTSD, 85 patients (15%) had partial PTSD (i.e., scored between 24–33 on the IES-R), and 276 patients (48.25%) met the cut-off score for PTSD. Table 2 details the prevalence rates of PTSD by type of cardiac event.

**Table 2:** PTSD category by type of cardiac event.

**Cardiac event**	**PTSD category**	**Frequency (percentage) [95% CI]**
Any	No PTSD	211 (37%) [33 to 41%]
	Partial PTSD	85 (15%) [12 to 18%]
	PTSD	276 (48%) [44 to 52%]
Acute coronary syndrome	No PTSD	139 (36%) [31 to 41%]
	Partial PTSD	64 (17%) [13 to 21%]
	PTSD	182 (47%) [42 to 52%]
Heart valve repair/replacement	No PTSD	13 (31%) [18 to 41%]
	Partial PTSD	8 (19%) [9 to 34%]
	PTSD	21 (50%) [34 to 66%]
Other	No PTSD	126 (36%) [31 to 41%]
	Partial PTSD	51 (14%) [11 to 19%]
	PTSD	175 (50%) [44 to 55%]

### Relationship between metacognitive beliefs and PTSD severity

**Figure 1:**
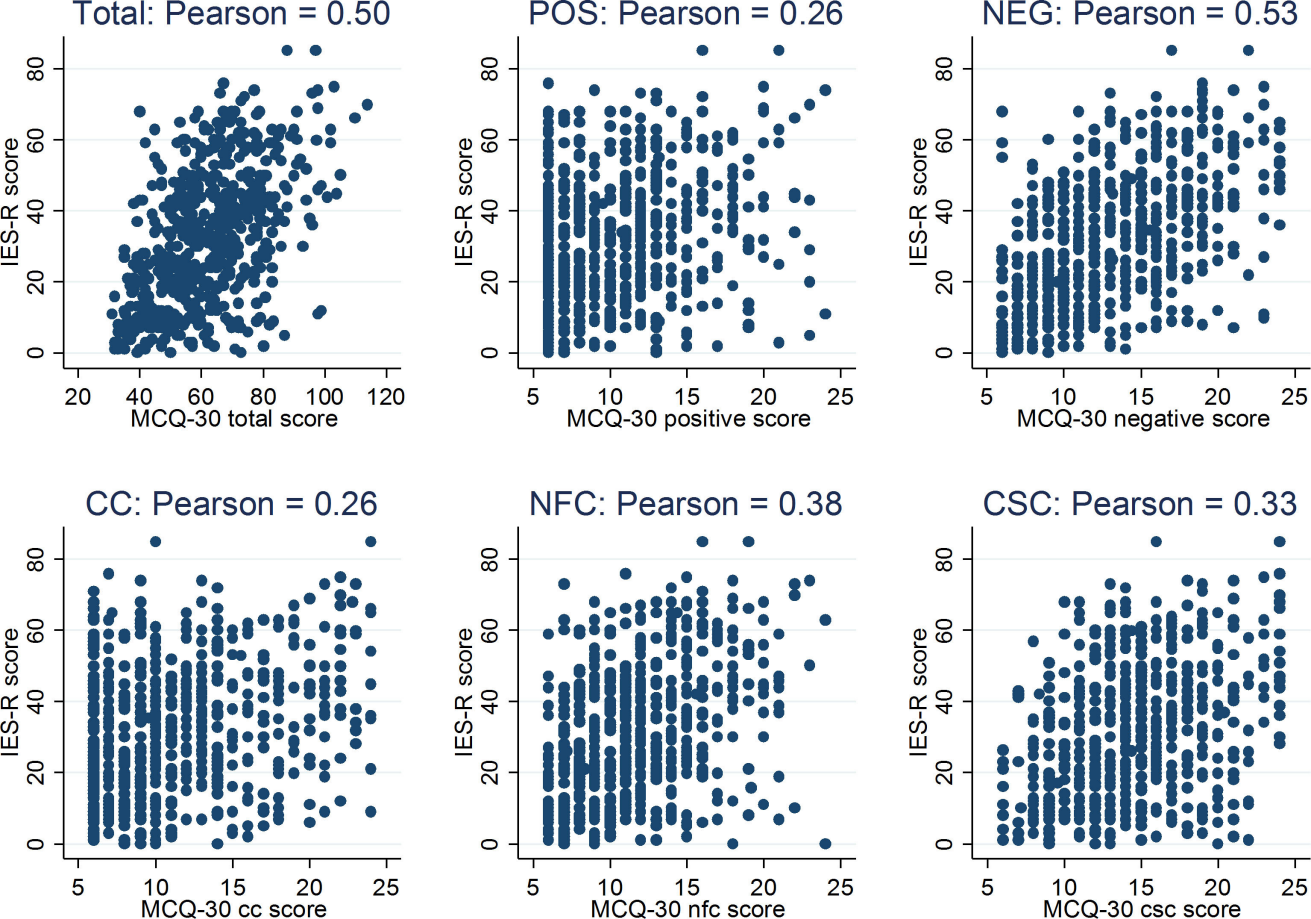
Scatter plots of MCQ-30 (and its subscales) and PTSD symptom severity (IES-R) scores.

Metacognitive beliefs were positively associated with symptoms of PTSD (as measured by the IES-R total score), such that greater dysfunctional metacognitive beliefs were associated with increased symptoms, see Figure 1. Correlations were moderate for the MCQ total, r = 0.50, *p* < 0.001, and negative beliefs regarding uncontrollability and danger, r = 0.53, *p* < 0.001. Weaker correlations were noted between PTSD severity and need for control, r = 0.38, *p* < 0.001, cognitive confidence r = 0.26, *p* < 0.001, cognitive self-consciousness, r = 0.33, *p* < 0.001, and positive metacognitive beliefs, r = 0.26, *p* < 0.001.

### Metacognitive predictors of PTSD category

Relative risk ratios and confidence intervals for the MCQ30 subscale and total scores analyzed as predictors of PTSD case-ness are summarized in Table 3. After adjusting for covariates, each subscale of the MCQ and the total score were significantly related to being classified as PTSD rather than no PTSD. The MCQ-neg subscale was most strongly associated with being in the PTSD category (as compared to the no PTSD category); for a one-unit increase in MCQ neg, the likelihood of being in the PTSD category increased by 30%, whilst the likelihood of being in the partial PTSD category increased by 16%. The next largest association was for MCQ need for control, followed by MCQ cognitive self-consciousness. Inspection of the rescaled RR values shows that the total MCQ score had a slightly higher risk ratio than the MCQ-neg subscale when their respective scores were rescaled between 0–100.

**Table 3:** Relative risk ratio and confidence intervals for metacognitive beliefs as predictors of PTSD.

**MCQ scale**	**IES class**	**Relative risk ratio**	**95% CI**	**P**	**Rescaled relative risk**	**95% CI**
MCQ total	Partial PTSD	1.04	1.02 to 1.06	< 0.001	1.03	1.02 to 1.05
	PTSD	1.08	1.06 to 1.10	< 0.001	1.06	1.05 to 1.08
MCQ positive	PartialPTSD	1.08	1.02 to 1.15	0.015	1.01	1.00 to 1.03
	PTSD	1.12	1.07 to 1.18	< 0.001	1.02	1.01 to 1.03
MCQ Neg-ud	Partial PTSD	1.16	1.08 to 1.25	< 0.001	1.03	1.01 to 1.04
	PTSD	1.30	1.23 to 1.38	< 0.001	1.05	1.04 to 1.06
MCQ cc	Partial PTSD	1.04	0.98 to 1.10	0.170	1.01	1.00 to 1.02
	PTSD	1.11	1.06 to 1.16	< 0.001	1.02	1.01 to 1.03
MCQ nfc	Partial PTSD	1.06	0.99 to 1.15	0.111	1.01	1.00 to 1.02
	PTSD	1.22	1.15 to 1.29	< 0.001	1.04	1.03 to 1.05
MCQ csc	Partial PTSD	1.13	1.06 to 1.21	< 0.001	1.02	1.01 to 1.03
	PTSD	1.18	1.12 to 1.24	< 0.001	1.03	1.02 to 1.04

MCQ, metacognitions questionnaire; positive, positive metacognitive beliefs; neg ud, negative metacognitive beliefs concerning uncontrollability and danger; cc, cognitive confidence; nfc, need for control; csc, cognitive self confidence.

### Metacognitive predictors of PTSD symptom severity

Summary statistics for the linear regression models are presented in Table 4. The pre-specified covariate set explained 14% of the variance in IES score, and entering each of the MCQ30 subscales and the MCQ total individually alongside the covariates resulted in a statistically significant increase in the variance explained. Models including the MCQ-30 total score or the negative subscale (uncontrollability and danger) accounted for the largest amount of overall variance in the IES-R (30.8% and 31.9% respectively). Compared to the covariates alone, adding negative beliefs concerning uncontrollability and danger (ud) into the model explained an additional 18.1% of variance (i.e. 31.9% compared to 13.8%) whilst need for control (nfc) explained an additional 10.6%, and cognitive self-consciousness (csc) 7.8%. Adding the MCQ positive beliefs and cognitive confidence (cc) subscales to the covariates led to smaller increments in variance accounted for (4.3% and 3.9% respectively).

**Table 4:** Adjusted R squared values for regression models of IES-R using covariates alone (demographic and heath factors), and covariates plus each of the MCQ-30 scores (total and subscale scores) individually.

	**Adjusted R^2^**	**Significant improvement over covariates only (LR test)**
Covariates only	0.138	Reference
MCQ total score	0.308	< 0.001
MCQ positive	0.181	< 0.001
MCQ neg ud	0.319	< 0.001
MCQ cc	0.177	< 0.001
MCQ nfc	0.244	< 0.001
MCQ csc	0.216	< 0.001

MCQ, metacognitions questionnaire; neg ud, negative beliefs/uncontrollability and danger, cc, cognitive confidence, nfc, need for control; csc, cognitive self confidence.

The results of the stepwise regression analysis to determine the optimal subset of predictors are summarized in Table 5. Both forward entry and backward elimination resulted in the same two MCQ subscales remaining in the equation; negative beliefs (ud) and need for control (nfc), along with the three covariates; age, sex, and number of comorbid disorders.

**Table 5:** Summary of optimal stepwise regression model for IES-R scores.

**Variable**	**Coefficient**	**95% CI**	***P***
MCQ nfc	0.72	0.33 to 1.11	< 0.001
MCQ neg ud	1.56	1.21 to 1.91	< 0.001
Age	−0.26	−0.38 to −0.13	< 0.001
Sex	3.86	1.09 to 6.62	0.006
Previous cardiac event	−0.77	−3.44 to 1.89	0.57
Number comorbidities	1.08	0.40 to 1.77	0.002
Previous anxiety	1.48	−1.95 to 4.90	0.40
Previous depression	−0.31	−3.73 to 3.11	0.86

Adjusted R^2^ = 0.33. MCQ, metacognitions questionnaire; nfc, need for control; neg ud, negative beliefs/uncontrollability and danger.

## Discussion

Among heart disease patients attending CR and scoring at least mild anxiety/depression symptoms under routine screening with the HADs, we found high mean PTSD symptom scores and a high prevalence of PTSD cases based on IES-R thresholds. There was no evidence of higher PTSD being associated with type of cardiac event, with rates similar across acute coronary syndrome, heart-valve repair/replacement or other event classifications. PTSD symptom severity was positively correlated with each metacognitive subscale, with beliefs concerning the uncontrollability and dangerousness of thoughts (MCQ-neg UD) making the strongest contribution individually after controlling for covariates.

Rates of PTSD were high, with 48% of patients meeting the threshold for PTSD and a further 15% partial PTSD. Thus, almost two-thirds of the sample met criteria for symptoms of potential “clinical concern” as defined by IES-R cut-off scores. These rates are higher than those reported previously of up to 35% (5), which is probably accounted for by the preselection in the current sample of patients who have at least mild anxiety/depression on the HADS. These results highlight the importance of assessing specific psychological morbidity and PTSD in particular in patients showing elevated anxiety/depression scores. All metacognitive subscales were associated with increased likelihood of a PTSD classification, with uncontrollability and danger the strongest predictor. These results are consistent with metacognitive theory, where beliefs about loss of control and harmfulness of cognition in particular, are core factors behind psychological vulnerability and poor adaptation to stress (33). Taking the raw MCQ-30 subscale scores, for every unit increase in MCQ negative belief (uncontrollability and danger) the odds of being in the PTSD group increased by 30%, whilst the likelihood of being in the partial PTSD group increased by 16%. Thus, small increments in MCQ uncontrollability and danger were associated with large increments in the odds of having PTSD. The rescaled MCQ scores allow a level comparison of the relative effects of total MCQ score against the MCQ subscales and show that total score has a risk ratio (RR) that is only slightly above the RR of uncontrollability and danger.

In testing for an optimal set of independent predictors for PTSD symptom severity we found that MCQ-neg (uncontrollability and danger) and MCQ need for control made significant unique contributions. There were also contributions from other covariates. In sum, greater PTSD symptom severity was associated with being female, younger, having more comorbidities, and reporting elevated MCQ-neg and MCQ-nfc scores. This pattern of results is potentially informative in developing a profile of heart disease patients with elevated HADS scores who are likely to be suffering from post-traumatic stress.

The potential limitations of the current study method should be considered in interpreting the findings. We did not use diagnostic interviews in screening for PTSD as these are expensive and not routinely available in cardiac services, instead we relied on the IES-R for detecting symptoms and making clinically relevant cut-offs for cases. The IES-R shows good to excellent ability to identify PTSD cases when validated against DSM-IV or DSM-V criteria (27, 31), but we cannot rule-out that PTSD in the current sample may differ in some respects from PTSD defined by diagnostic interview. The current sample includes patients with scores of at least 8 on a HADS subscale at initial screening, and therefore the results should not be interpreted in the context of all CHD patients. Our sample is likely to give higher estimates in comparison with the overall proportion of CHD patients with PTSD. However, the data show that for those with at least mild anxiety and depression symptoms, there is a high prevalence of PTSD symptoms meeting thresholds of probable clinical importance.

The identification of PTSD symptoms in CHD and when implementing CR is valuable if interventions are going to be sensitive to and effectively tailored to meet the psychological needs of patients. In particular, PTSD may impact on outcomes and attendance at CR and may itself be affected by the components of CR. For example, attending CR may increase the severity of PTSD symptoms such as intrusive memories which are highly distressing for some patients. An implication is that assessment of PTSD symptoms would contribute to adjusting delivery of CR to accommodate the psychological needs of the individual.

Whilst we cannot infer causality from the current cross-sectional data, the results demonstrate that specific metacognitions are associated with a greater likelihood of having PTSD in patients with cardiac disease. The metacognitions identified as associated with highest trauma symptomatology correspond to those considered to have a central causal/maintaining role in the metacognitive model (15). The results support the potential application of the model to understanding and treating traumatic stress symptoms in cardiac patients. Specifically, the results are consistent with the idea that interventions that modify dysfunctional beliefs concerning the uncontrollability and dangerous effects of thoughts could have beneficial effects and reduce PTSD symptoms. In accordance with this, the addition of metacognitive therapy to CR, which focuses on modifying such beliefs has been found to be associated with significant improvements in anxiety, depression and PTSD symptoms in randomized trials of patients with CHD (22, 23).

In conclusion, the present findings have several potential implications for the management of psychological symptoms in CHD. First, they highlight a need for more specific assessment of PTSD in patients showing anxiety or low mood. Second, they suggest that general approaches to the management of anxiety/depression symptoms in CR may not meet the needs of a large proportion of patients who have underlying PTSD. Third, the results are consistent with application of the metacognitive model and metacognitive therapy aimed at modifying risk factors such as uncontrollability and danger metacognitions that are linked to both PTSD and other psychological morbidities. It appears that management of psychological symptoms in CR might take two different routes; (i) introduce specific PTSD-focused treatment methods for those service users that need them or; (ii) adopt a transdiagnostic treatment approach such as metacognitive therapy that is designed to target universal factors associated with multiple morbidities including PTSD. Metacognitive therapy is effective in mental health settings in treating PTSD (34) and within CR the inclusion of MCT is associated with significant improvements in HADs anxiety/depression and PTSD symptoms (22, 23). The results of the present study add further support for improving psychological outcomes in CHD by targeting metacognition.

## Data availability statement

The datasets presented in this article are not readily available because participants did not grant permission for public repository of their data. Requests to access the datasets should be directed to the study sponsor: Greater Manchester Mental Health and Social Care NHS Trust. Contact: researchoffice@gmmh.nhs.uk.

## Ethics statement

Ethical approval was obtained from the Preston Research Ethics Committee (REC Reference 14/NW/0163) and from the North West—Greater Manchester West Research Ethics Committee (REC Reference 16/NW/0786). The patients/participants provided their written informed consent to participate in this study.

## Author contributions

AW: study conception, grant writing, data analysis, interpretation, and manuscript writing. CH: statistical analysis plan, data analysis, and manuscript writing. DR: grant writing, data-analysis, and manuscript writing. LC: manuscript writing, data analysis, and interpretation. All authors contributed to the article and approved the submitted version.
